# Polypharmazie bei akuten und chronischen Nierenerkrankungen

**DOI:** 10.1007/s00108-023-01634-7

**Published:** 2023-12-18

**Authors:** Roxana Manaila, Andrea Huwiler

**Affiliations:** Institut für Pharmakologie, Universität Bern, Inselspital, INO-F, 3010 Bern, Schweiz

**Keywords:** Akute Nierenschädigung, Unerwünschte Arzneimittelwirkungen, Arzneimittelinteraktionen, Therapieadhärenz, Reduktion der Anzahl verschriebener Medikamente, Acute kidney injury, Drug-related side effects and adverse reactions, Drug interactions, Therapeutic adherence, Deprescribing

## Abstract

Die Prävalenz der chronischen Nierenerkrankung („chronic kidney disease“ [CKD]) ist in den letzten Jahrzehnten stetig angestiegen. Die CKD ist eine graduell progressive Erkrankung, die mit mehreren Begleiterkrankungen assoziiert ist, beispielsweise mit kardiovaskulären Erkrankungen, Bluthochdruck, Anämie, Störungen des Mineral- und Knochenstoffwechsels, Elektrolytveränderungen und Störungen des Säure-Basen-Haushalts. Alle diese Begleiterkrankungen erfordern eine adäquate Medikation. Daher haben Patienten mit CKD ein hohes Risiko der Polypharmazie, die als Behandlung mit mehr als 5 Arzneimitteln täglich definiert ist. Polypharmazie geht mit einem stark erhöhten Risiko unerwünschter Arzneimittelwirkungen und schwerer Arzneimittelinteraktionen einher, die zu erhöhter Morbidität und Mortalität führen, wenn sie nicht gut kontrolliert werden und wenn die einzelnen Dosen während des Fortschreitens der CKD nicht entsprechend dem Nierenfunktionsabfall angepasst werden. Daher sollten mehrere Aspekte der Medikation beachtet und konstant überprüft werden. Der vorliegende Beitrag zeigt die Probleme auf, die sich aus einer inadäquaten Polypharmazie bei CKD ergeben; zu diesen zählen unerwünschte Arzneimittelwirkungen und -interaktionen, die Komplexität der Therapieschemata, die Therapiebelastung und die Nichteinhaltung der Therapie. Zudem werden die wichtigsten Schritte zur Identifikation einer inadäquaten Polypharmazie diskutiert, wodurch sich Komplikationen vermeiden lassen und der Nutzen der Medikation erhöht werden kann. Zuletzt wird auf die Polypharmazie bei akuter Nierenschädigung eingegangen.

## Abstract (en)

The prevalence for chronic kidney disease (CKD) has steadily increased over the past decades. It is a gradually progressive disease that is associated with several comorbidities including cardiovascular diseases, hypertension, anemia, disorders of bone and mineral metabolism, electrolyte imbalance and acid-base abnormalities. All these comorbidities require adequate medication. Therefore, patients with CKD have a high risk for polypharmacy, which is defined as five or more medications daily. Polypharmacy causes a greatly increased risk for adverse drug effects and severe drug-drug interactions, which if not closely controlled and the individual doses adapted to the decreased renal function during the progression of the CKD, can result in increased morbidity and mortality. Therefore, several aspects of the medication need to be considered and constantly addressed. This article summarizes the problems arising from inadequate polypharmacy in CKD patients, including undesired adverse drug effects, drug interactions, the complexity of medication plans, treatment burden and nonadherence to the treatment. Furthermore, the most important steps to identify patients with inadequate polypharmacy are discussed, whereby complications can also be avoided and the benefits of the medication can be increased. Finally, the polypharmacy in acute kidney injury is dealt with.

Die am weitesten verbreitete Definition von Polypharmazie ist eine strikt numerische: die tägliche Einnahme von > 5 Arzneimitteln ohne weitere Wertung des Nutzens [1]. Obwohl häufig mit einem negativen Vorurteil verbunden, kann die Verwendung von > 5 Arzneimitteln durchaus angezeigt sein. Gerade beim gleichzeitigen Auftreten mehrerer chronischer Erkrankungen kann durch gezielte Therapie der einzelnen Erkrankungen die Morbidität gesenkt werden.

Da eine zunehmende Multimorbidität besonders im Alter auftritt, ist auch besonders diese Patientengruppe sehr stark von Polypharmazie betroffen. Die durchschnittliche Lebenserwartung in der industrialisierten Welt ist in den letzten Jahrzehnten stark angestiegen. Daher überrascht es nicht, dass das Ausmaß der Polypharmazie in diesem Zeitraum massiv zugenommen hat. Studien in den USA zeigen, dass der Anteil der Patienten über 65 Jahre, die mehr als 5 Arzneimittel einnehmen, zwischen 1999 und 2012 von 24 % auf 39 % angestiegen ist [2]. Ähnlich ergab eine Studie aus Großbritannien, dass sich die Anzahl älterer Menschen, die 5 oder mehr Arzneimittel einnehmen, zwischen 1994 und 2011 von 12 % auf fast 50 % vervierfacht hat [3].

Die Hauptrisiken und Probleme, die aus einer Polypharmazie entstehen, sind die erhöhte Wahrscheinlichkeit schwerwiegender Nebenwirkungen, Arzneimittelinteraktionen, komplizierte Therapieschemata, die zur Nichteinhaltung der Therapie führen, eine Überdiagnose und Überbehandlung ohne sichtbaren Nutzen und nicht zuletzt erhöhte Gesundheitskosten, die nicht immer von Krankenversicherungen übernommen werden (Abb. 1). Am alarmierendsten ist jedoch eine berichtete Assoziation der Polypharmazie mit einer erhöhten Mortalität [4], wobei die Kausalität dieser Beziehung unklar bleibt. Die Assoziation unterstreicht dennoch die Notwendigkeit, nach Ansätzen in der Gesundheitsversorgung zu suchen, mit denen ein optimales Gleichgewicht zwischen Risiko und Nutzen bei der Verschreibung von Medikamenten erreicht werden kann. Selbst wenn wir die Kausalität akzeptieren würden, könnten die nachteiligen Folgen der Polypharmazie durch eine Vielzahl potenzieller klinischer Vorteile ausgeglichen werden, was bedeutet, dass die Kausalität nicht zwangsläufig einen inhärenten Schaden beweist.

**Figure 1:**
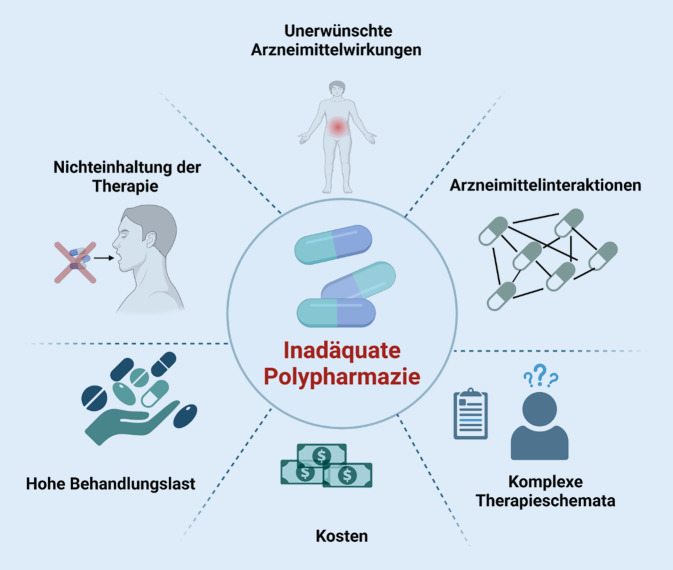

Im Gegensatz dazu konnten andere Studien keine Korrelation zwischen Polypharmazie und erhöhten Krankenhausaufnahmen finden. Diese Studien deuten vielmehr darauf hin, dass Polypharmazie angemessen sein kann [5]. Es wäre daher angebracht, den Begriff Polypharmazie zukünftig in „adäquate Polypharmazie“ und „inadäquate Polypharmazie“ zu spezifizieren; dies erlaubt die Unterscheidung zwischen der Verschreibung von „vielen“ Medikamenten, was nicht unbedingt negativ ist, weil die potenziellen Vorteile die potenziellen Schäden überwiegen, und „zu vielen“ Medikamenten, was den Patienten einem Risiko für nachteilige Gesundheitsauswirkungen aussetzt. Der vorliegende Übersichtsbeitrag fast die wichtigsten Aspekte zur Polypharmazie bei chronischen und akuten Nierenerkrankungen zusammen.

## Problematik der Polypharmazie bei Patienten mit chronischer Nierenerkrankung

Bei Patienten mit chronischer Nierenerkrankung („chronic kidney disease“ [CKD]) erfordert die Polypharmazie spezielle Aufmerksamkeit und ein sorgfältiges Management, da die Nierenfunktion beeinträchtigt ist und viele Medikamente über die Nieren ausgeschieden werden.

Eine CKD ist durchaus eine häufige Erkrankung und betrifft weltweit 10 % (in den USA bereits 16 %) der erwachsenen Bevölkerung mit einer stetig steigenden Prävalenz [6]. In absoluten Zahlen wurden im Jahr 2017 weltweit 697,5 Mio. Fälle von CKD registriert [6] und 1,2 Mio. Menschen starben an einer CKD. Der Hauptgrund für diesen Anstieg von CDK liegt in einem gleichzeitigen Anstieg der wichtigsten drei Risikofaktoren, nämlich Diabetes, Fettleibigkeit und Bluthochdruck, bedingt durch eine allgemein veränderte Lebensweise. Eine CKD entwickelt sich stufenweise progressiv und ist mit einer Reihe von Komplikationen und Begleiterkrankungen verbunden, beispielsweise mit kardiovaskulären Erkrankungen, Anämie, Störungen des Mineral- und Knochenstoffwechsels, Elektrolytveränderungen und Störungen des Säure-Basen-Haushalts. Daher sind Patienten mit CKD sehr stark auf Polypharmazie angewiesen. Arzneimittel, die bei CKD und Begleiterkrankungen häufig eingenommen werden und die eine besondere Kontrolle und Dosisanpassung erfordern, sind in Tab. 1 zusammengefasst.

**Table 1:** 

Häufig verschriebene Arzneimittel, die eine Dosisanpassung oder ein Absetzen bei Patienten mit chronischer Nierenerkrankung erfordern^a^					Arzneimittel mit nephrotoxischer Wirkung
Antidiabetika	Antibiotika/Virostatika	Herz-Kreislauf-Medikamente	Analgetika	Andere	
Acarbose Exenatid Glibenclamid Gliclazid Glimepirid Glipizid Insulin Metformin^b^ Saxagliptin Sitagliptin Vildagliptin	Famciclovir Trimethoprim^c^ Valaciclovir	Apixaban Dabigatran Digoxin Fenofibrat Rivaroxaban Sotalol Spironolacton	Gabapentin Opioide NSAR Pregabalin	Allopurinol Benzodiazepine Colchicin Denosumab Lithium	Aminoglykoside Calcineurininhibitoren Gadolinium Lithium NSAR Kontrastmittel

*GFR* glomeruläre Filtrationsrate, *NSAR* nichtsteroidale Antirheumatika

^a^Diese Liste ist nicht abschließend

^b^Metformin wird nicht empfohlen bei einer GFR < 30 ml/min pro 1,73 m^2^

^c^Trimethoprim kann den Serumkreatininspiegel erhöhen, hat aber keine Wirkung auf die GFR

### Unerwünschte Arzneimittelwirkungen

Mit jedem zusätzlichen Medikament steigt das Risiko unerwünschter Arzneimittelwirkungen (UAW), die die Lebensqualität beeinträchtigen und zu weiteren gesundheitlichen Problemen führen können. Polypharmazie sollte daher vermieden werden, wenn die Nebenwirkungen der Medikamente schwerwiegender sind als die zugrunde liegenden Gesundheitsprobleme.

Die GFR muss engmaschig kontrolliert, die Anwendung von Arzneimitteln konstant überprüft werden

UAW bei Patienten mit eingeschränkter renaler Clearance können lebensbedrohlich sein, verlängern eine Hospitalisierungsdauer und erhöhen konsequenterweise die Gesundheitskosten. In einer multizentrischen retrospektiven Kohortenstudie an > 100.000 hospitalisierten Patienten mit CKD wurde über einen Zeitraum von 20 Monaten die Inzidenz und Schwere von UAW untersucht, die durch Arzneimittel verursacht wurden, welche renal eliminiert werden oder ein nephrotoxisches Potenzial besitzen [7]. Die Daten zeigen, dass 10 % der Patienten UAW erlitten, wobei 4,5 % lebensbedrohlich waren [7]. Dabei waren Antibiotika, Analgetika und kardiovaskuläre Medikamente am häufigsten an der Verursachung vermeidbarer UAW beteiligt. Die meisten UAW führten zu einer akuten Verschlechterung der Nierenfunktion. Der häufigste Fehler lag in einer falschen Dosierung (86 %) oder falschen Einnahmehäufigkeit (12 %), gefolgt von der Verabreichung nephrotoxischer Medikamente bei steigendem Kreatinin. Unter allen UAW wurden 9 von 10 als vermeidbar angesehen [7, 8]. Darüber hinaus stieg die Wahrscheinlichkeit für UAW exponentiell mit abnehmender Nierenfunktion, wobei mehr als zwei Drittel der UAW bei Patienten mit terminaler Nierenerkrankung auftraten. Auch hatten Patienten mit niedrigem Serumalbuminspiegel ein erhöhtes UAW-Risiko, was durch einen Anstieg des freien Anteils der sonst albumingebundenen Arzneimittel im Plasma erklärt werden kann. Zusätzlich hatten diejenigen Patienten, die UAW entwickelten, einen erhöhten Serumspiegel von C‑reaktivem Protein (CRP), und sowohl der CRP-Wert als auch Gefäßerkrankungen wurden als signifikante Prädiktoren für die Entwicklung von UAW vorgeschlagen [7].

In einer kürzlich publizierten prospektiven Kohortenstudie aus Frankreich (Chronic Kidney Disease – Renal Epidemiology and Information Network [CKD-REIN], registriert auf ClinicalTrials.gov unter NCT03381950) wurden > 3000 Patienten eingeschlossen, die eine bestätigte Diagnose einer mäßigen oder fortgeschrittenen CKD, das heißt eine glomeruläre Filtrationsrate (GFR) < 60 ml/min pro 1,73 m^2^ hatten und nicht dialysepflichtig oder im Zustand nach Transplantation waren [9]. In der Studie wurden das Ausmaß und die Vielfalt der UAW in der ambulanten Kohortengruppe untersucht, zudem die Ursächlichkeit, Vermeidbarkeit sowie Faktoren, die mit UAW und unmittelbarem therapeutischem Management in Verbindung standen. Die Hauptbotschaft dieser Studie ist ebenso, dass UAW häufig vorkommen, oft schwerwiegend sind (150 UAW bei 125 Teilnehmern, wobei 16 von ihnen direkt oder indirekt zum Tod führten) und sich potenziell vermeiden lassen (32 % der Fälle). Die drei am häufigsten verschriebenen Arzneimittelklassen waren für fast 40 % der UAW verantwortlich, genauer Inhibitoren des Renin-Angiotensin-Aldosteron-Systems (RAAS), Antithrombotika und Diuretika. Die für die UAW hauptverantwortlichen Arzneimittel waren in der Regel nicht direkt nephrotoxisch; die meisten UAW resultierten aus einer reduzierten renalen Clearance. Auch diese Studie belegte die Notwendigkeit, die GFR engmaschig zu kontrollieren und die Anwendung oder Dosierung von Arzneimitteln konstant zu überprüfen und anzupassen [9].

### Arzneimittelinteraktionen

Potenzielle Arzneimittelinteraktionen („potential drug–drug interactions“ [pDDI]) können auf verschiedenen Ebenen auftreten und Veränderungen sowohl in der Pharmakodynamik als auch in der Pharmakokinetik von Arzneimitteln auslösen. Arzneimittelinteraktionen können neue Beschwerdebilder hervorrufen und damit weitere Verschreibungen nötig machen. Die Polypharmazie ist ein Hauptfaktor für pDDI, die mit der Anzahl der verschriebenen Medikamente ansteigen und bei > 8 Arzneimitteln 100 % erreichen [10].

Um pDDI zu identifizieren, wurden verschiedene Computerprogramme entwickelt und auch miteinander verglichen [11, 12]. Dazu gehören Micromedex Drug-Reax® (Merative L.P., Ann Arbor, CA, USA; http://www.micromedex.com), Facts and Comparisons® (Wolters Kluwer, Alphen aan den Rijn, Niederlande; http://www.factsandcomparisons.com), Pharmavista (HCl Solutions AG, Bern, Schweiz; http://www.pharmavista.ch), Epocrates Rx® (Epocrates Inc., San Mateo, CA, USA; http://www.epocrates.com), mediQ® (Psychiatrische Dienste Aargau AG, Windisch, Schweiz; https://www.mediq.ch), Lexicomp® (Wolters Kluwer, Alphen aan den Rijn, Niederlande; http://www.lexi.com) und Drug Interaction Checker (Drugsite Ltd., Dallas, TX, USA; http://www.drugs.com). Lexicomp® beispielsweise gilt als leistungsfähig und wurde als hochsensitiv und spezifisch beschrieben. Es klassifiziert pDDI nach ihrer klinischen Relevanz in 5 Typen:

- Typ A: keine bekannten Interaktionen
- Typ B: geringe oder milde Interaktionen
- Typ C: moderate oder signifikante Interaktionen
- Typ D: starke oder schwerwiegende Interaktionen
- Typ X: kontraindiziert oder Kombination vermeiden

### Komplexe Therapieschemata

Je höher die Anzahl der einzunehmenden Arzneimittel, umso komplizierter werden die Therapieschemata. Patienten müssen sich an verschiedene Einnahmezeiten und Dosierungen halten sowie mögliche Wechselwirkungen beachten, nicht nur zwischen Arzneimitteln, sondern auch mit Nahrung und Getränken. Dies kann sehr belastend oder gar überfordernd sein, insbesondere bei älteren Patienten mit kognitiven Beeinträchtigungen. Bei komplexen Therapieschemata ist eine sorgfältige Planung und Überwachung essenziell. Die Verwaltung der Medikamente kann durch Medikamentenlisten, Pillendispenser, Apps mit Erinnerungsweckern oder spezielle Arzneimittelverpackungen erleichtert werden. Zusätzlich ist eine gute Patientenaufklärung entscheidend, damit Medikamente korrekt eingenommen werden und der bestmögliche Nutzen erreicht werden kann.

### Hohe Behandlungslast

Polypharmazie und die damit verbundene Pillenlast können zu einer „Behandlungsbelastung“ für den Patienten führen. Patienten mit CKD, insbesondere solche mit einer Nierenersatztherapie, tragen eine hohe tägliche Pillenlast. Die Deutsche CKD-Studie (German Chronic Kidney Disease Study) wertete Daten von insgesamt 5217 Erwachsenen mit einer errechneten GFR (eGFR) von 30 bis 60 ml/min pro 1,73 m^2^ oder einer eGFR > 60 ml/min pro 1,73 m^2^ und offener Proteinurie (> 500 mg/Tag) aus und fand eine Prävalenz für Polypharmazie von fast 80 %, mit Werten von 62 % bei Patienten im CKD-Stadium G1 bis 86 % bei Patienten im CKD-Stadium G3b [13]. Eine ähnlich hohe Medikamentenbelastung und Prävalenz für Polypharmazie wurde auch in anderen Studien aus Frankreich, den Niederlanden und den USA vermeldet [14–16].

Patienten mit chronischer Nierenerkrankung haben eine hohe tägliche Pillenlast

Interessanterweise wurde berichtet, dass sich Kombinationspräparate und Formulierungen mit verlängerter Freisetzung und geringerer Dosierungshäufigkeit als wirksam hinsichtlich einer Verringerung der Pillenbelastung erwiesen haben und auch kosteneffizienter sind [17]. Ein Beispiel für ersteren Ansatz sind Tabletten, die zwei oder drei Klassen von blutdrucksenkenden Wirkstoffen kombinieren, anstelle von Tabletten für jede einzelne Substanz. Ein Beispiel für letzteren Ansatz ist die Verwendung von 60 mg-Gliclazid-Tabletten mit modifizierter Freisetzung 1‑mal täglich anstelle von 30 mg-Tabletten 2‑mal täglich zur Behandlung von Diabetes.

### Nichteinhaltung der Therapie

Eine hohe Medikamentenbelastung führt häufig zur Nichteinhaltung („non-adherence“) der Therapie durch den Patienten. Daten deuten darauf hin, dass 50–80 % der Patienten ihre Medikamente nicht wie verschrieben einnehmen [18], und dies gilt auch für Patienten mit CKD. Insgesamt hat etwa ein Drittel der Patienten mit CKD eine schlechte Compliance [19]. Nicht nur die hohe Pillenbelastung, sondern auch die Komplexität der Therapieschemata beeinträchtigt die Einhaltung der Therapie. Es erscheint vernünftig, die Medikamentenschemata zu vereinfachen und die Anzahl der Pillen zu reduzieren, um die Einhaltung zu verbessern. Aus einer Umfrage unter 54 Patienten mit chronischer Dialyse ging hervor, dass unzureichende Verschreibung, ein fehlendes Transportmittel zum Dialyseort und die Medikamentenkosten wesentliche Ursachen für die Nichteinhaltung in dieser Gruppe sind [20]. Die Patienten gaben an, ihre Medikamente zu kennen, die Gründe zu kennen, warum diese Medikamente verschrieben wurden, und ausreichend Gelegenheiten zu haben, mit ihren Ärzten über ihre Medikamente zu sprechen. Eine mangelnde Patientenaufklärung scheint daher keine wichtige Ursache für die schlechte Therapieeinhaltung zu sein. Es wurde ein deutlicher Zusammenhang zwischen einer schlechten Therapieeinhaltung und einem erhöhten Auftreten von UAW, wie Hypoglykämie, Stürzen, Hypotonie und Hyperkaliämie, bei Patienten mit CKD berichtet [21].

## Maßnahmen bei Patienten mit chronischer Nierenerkrankung und Polypharmazie

### Überwachung der Nierenfunktion

Die Nierenfunktion sollte regelmäßig überwacht werden, um Veränderungen im Verlauf der Erkrankung zu erfassen. Die Berechnung der eGFR anhand der Serumkreatininkonzentration kann mithilfe der Cockcroft-Gault-Formel durchgeführt werden [22]. Sie basiert auf dem Serumkreatininspiegel, dem Alter des Patienten, dem Geschlecht und dem Körpergewicht. Eine noch genauere Schätzung der eGFR erhält man durch die Modification-of-Diet-in-Renal-Disease(MDRD)-Gleichung [23] oder die CKD-Epidemiology-Collaboration(CKD-EPI)-Gleichung [24].

### Arzneimittelauswahl

Bei der Auswahl von Medikamenten für Patienten mit Nierenerkrankung ist Vorsicht geboten, da einige Medikamente die Nierenfunktion weiter verschlechtern können oder schwere Nebenwirkungen verursachen. Man spricht auch von potenziell inadäquaten Medikamenten (PIM).

Es existieren verschiedene PIM-Listen, die inadäquate Arzneimittel auflisten. Internationale PIM-Listen sind aufgrund unterschiedlicher Marktgegebenheiten und Verschreibungspraktiken schwer auf nationaler Ebene umsetzbar. Die Beers-Kriterien der American Geriatrics Society sind eine PIM-Liste, die inadäquate Arzneimittel speziell für ältere Patienten enthält. Eine für Deutschland relevante PIM-Liste ist die PRISCUS-Liste, die eine praxistaugliche Zusammenstellung von Wirkstoffen und Wirkstoffgruppen enthält, die als potenziell inadäquat für ältere Menschen beurteilt werden. Die Liste informiert auch über Therapiealternativen und Maßnahmen, wenn der Einsatz potenziell inadäquater Wirkstoffe nicht zu umgehen ist. Auch die Fit-fOr-The Aged(FORTA)-Liste, erstellt von Experten der Medizinischen Fakultät Mannheim der Universität Heidelberg, ist eine PIM-Liste speziell für die Geriatrie, die nicht nur von Substanzen abrät, sondern bestimmte für ältere Patienten besonders geeignete Arzneimittel ausdrücklich empfiehlt.

Bei CKD und Polypharmazie sind Medikamente mit guter Nierenverträglichkeit vorzuziehen

Bei Patienten mit CKD und Polypharmazie sind solche Medikamente vorzuziehen, die eine relativ gute Nierenverträglichkeit haben. Insbesondere sollten nephrotoxische Medikamente, wenn möglich, vermieden werden. Dazu gehören bestimmte Antibiotika, nichtsteroidale Antirheumatika (NSAR) und Kontrastmittel für bildgebende Verfahren (Tab. 2).

**Table 2:** 

Mechanismus der Nephrotoxizität	Arzneimittel
Arzneimittelinduzierte akute tubuläre Nekrose	Aminoglykoside Amphotericin B Vancomycin
Nephrotoxische akute interstitielle Nephritis	NSAR, Penicillin, Cephalosporin, Ciprofloxacin, Sulfonamide (inklusive Trimethoprim + Sulfamethoxazol, Furosemid, Bumetanid, Thiazide), Allopurinol, Omeprazol, Indinavir
Hämodynamisch vermitteltes nephrotoxisches akutes Nierenversagen	ACE-Hemmer, ARB, NSAR, Vasopressoren, Calcineurininhibitoren
Tubuläre Obstruktion	Indinavir, Tenofovir

*ACE* „angiotensin-converting enzyme“, *ARB* Angiotensin-II-Rezeptor-Blocker, *NSAR* nichtsteroidale Antirheumatika

In der Studie von Laville et al. [14] wurden die Prävalenz und Determinanten unangemessener Medikamentenverschreibungen, ob Kontraindikationen oder unangemessen hohe Dosen, in Bezug auf die Nierenfunktion in einer Kohorte von 3033 ambulanten Patienten mit CKD (eGFR 15–60 ml/min pro 1,73 m^2^) untersucht. Wie die Studie erneut bestätigte, war Polypharmazie bei nicht dialysepflichtigen Patienten mit CKD häufig und die Anzahl der verschriebenen Medikamente der Hauptfaktor für unangemessene Verschreibungen. Ein Anteil von 31 % der Patienten hatte mindestens ein kontraindiziertes Medikament verschrieben bekommen, und 35 % mindestens ein in Bezug auf ihre eGFR inadäquat hoch dosiertes Medikament.

In einer systematischen Übersicht über unangemessene Medikation bei Patienten mit CKD berichteten Dorks et al. [25] eine Prävalenz von bis zu 43 % bei nichthospitalisierten Patienten. Bei hospitalisierten Patienten waren die Raten der Nichtbeachtung der renalen Dosierung höher und reichten von 15 bis 74 %. Das am häufigsten verschriebene Medikament mit unangemessener Anwendung war Metformin, gefolgt von Allopurinol und Glibenclamid.

### Dosisanpassung

Bei Patienten mit CKD muss die Dosierung vieler Medikamente angepasst werden, da die Ausscheidung über die Nieren reduziert ist. Die Anpassung sollte in Absprache mit einem Arzt erfolgen, der die Nierenfunktion einberechnet. Zu beachten ist, dass bei älteren Patienten eine CKD oft übersehen wird, da eine verringerte Muskelmasse zu einer scheinbar normalen Serumkreatininkonzentration führen kann. Die meisten Richtlinien zur Anpassung der Medikamentendosis an die Nierenfunktion basieren auf der Cockcroft-Gault-Gleichung [22] oder den verbesserten MDRD- [23] und CKD-EPI-Gleichungen [24].

### Medikamentenreduktion

Eine Strategie zur Reduktion von Polypharmazie und suboptimaler Medikamentenanwendung ist die Reduktion der Medikamentenanzahl („deprescribing“). Die Medikamentenreduktion wurde als der Prozess des Ausschleichens, Beendens, Absetzens oder Zurückziehens von Medikamenten definiert, mit dem Ziel, Polypharmazie zu bewältigen und Ergebnisse zu verbessern. Da ältere Menschen häufig Polypharmazie erleben, haben viele Studien die Medikamentenreduktion bei dieser Patientengruppe untersucht. Zu den Schlüsselschritten bei der Medikamentenreduktion gehört eine umfassende Überprüfung der Medikation, um PIM für die Beendigung oder Dosisreduktion zu identifizieren und zu priorisieren, gefolgt von einem Plan, die Medikamente sicher abzusetzen, und einer Patientenüberwachung nach dem Absetzen. Ein Beispiel für solche Werkzeuge, die für ältere Patienten stationär validiert wurden, ist die Anwendung der Screening-Tool-of-Older-Persons’-Prescriptions(STOPP)-/Screening-Tool-to-Alert-to-Right-Treatment(START)-Kriterien. Obwohl diese Algorithmen, die bei älteren Patienten bereits verwendet werden, einen Rahmen für die Entwicklung neuer Programme zur Medikamentenreduktion speziell für Dialysepatienten bieten könnten, werden sie bisher nicht auf ambulante Hämodialysepatienten angewendet, weil sie für diese Indikation noch nicht validiert wurden [26].

### Patientenaufklärung und pharmazeutische Betreuung

Die Einhaltung der verschriebenen Medikation (Compliance) ist entscheidend für den Erfolg einer Therapie. Daher ist es wichtig, Patienten über die Bedeutung der Medikamenteneinnahme, der Dosierung und möglicher Nebenwirkungen aufzuklären. Dies erfordert eine gute Kommunikation nicht nur zwischen Patient und Arzt, sondern auch zwischen Patient und Apothekern. Der pharmazeutischen Betreuung durch den Apotheker kommt in der heutigen Zeit eine immer wichtigere Rolle zu. Sie beinhaltet direkte Unterstützung bei der Verwaltung der Arzneimittel, beispielsweise das Überprüfen der Medikamentenlisten und der richtigen Arzneimittel in der richtigen Dosis, das Füllen von Pillendispensern und Einstellen von Erinnerungsweckern und Apps. Auch die frühzeitige Erkennung von Nebenwirkungen gehört dazu. Mehrere Single-center-Studien mit kurzer Laufzeit an ambulanten Patienten, die eine Hämodialyse durchliefen, haben gezeigt, dass klinische Pharmazeuten medikamentenbezogene Probleme erkennen und lösen können, die Arzneimitteladhärenz verbessern, Informationen zu Medikamenten bereitstellen, das Bewusstsein für unangemessene Medikamentenverschreibungen schärfen, biochemische und therapeutische Reaktionen auf die Medikamententherapie verbessern und die Anzahl der Patienten erhöhen können, die den Standard der Versorgung erreichen [27].

## Polypharmazie bei akuter Nierenschädigung

Mehrere Studien weisen darauf hin, dass Polypharmazie auch direkt oder indirekt mit einer akuten Nierenschädigung („acute kidney injury“ [AKI]) in Verbindung steht. Die AKI ist ein häufiges Syndrom, insbesondere bei hospitalisierten Patienten, und ist unabhängig und stark mit einer erhöhten Morbidität und Mortalität assoziiert. Eine CKD erhöht das Risiko von AKI, und ein AKI-Ereignis erhöht wiederum die Wahrscheinlichkeit der nachfolgenden Entwicklung einer CKD [28], was die Notwendigkeit einer fortlaufenden Überwachung unterstreicht. Hausarztpraxen befinden sich in einer einzigartigen Position, um Personen mit erhöhtem AKI-Risiko zu identifizieren und potenziell modifizierbare Expositionen anzugehen, um das Auftreten einer AKI zu verhindern.

Eine chronische Nierenerkrankung erhöht das Risiko einer akuten Nierenschädigung und umgekehrt

Die Arzneimittel, die am häufigsten zu einer AKI führen, sind systemisch angewendete Antibiotika, Diuretika, RAAS-Hemmer, Antineoplastika und NSAR [28]. Angiotensin-converting-enzyme(ACE)-Hemmer und Angiotensin-II-Rezeptor-Blocker (ARB) verursachen eine reversible Verringerung des glomerulären Blutflusses, und die GFR kann bei Beginn der Behandlung abnehmen. Wenn die Reduktion innerhalb von 2 Monaten nach Beginn der Therapie weniger als 25 % beträgt, sollte der ACE-Hemmer oder ARB weitergeführt werden. Wenn die Reduktion der GFR mehr als 25 % unter dem Ausgangswert liegt, sollte der ACE-Hemmer oder ARB abgesetzt und eine Überweisung an einen Nephrologen in Erwägung gezogen werden [29]. Die Kombination aus ACE-Hemmer (oder ARB), Diuretikum und NSAR oder Cyclooxygenase(COX)-2-Inhibitor (außer niedrig dosierter Acetylsalicylsäure) kann zu akutem Nierenversagen führen („Dreifachschlag“), insbesondere wenn ein Volumenmangel oder eine CKD vorliegt. Zur Vermeidung ist es entscheidend sicherzustellen, dass Personen, die ACE-Hemmer oder ARB zusammen mit Diuretika einnehmen, sich bewusst sind, dass sie mit ihrem Hausarzt oder Apotheker die Notwendigkeit einer angemessenen Schmerzlinderungsmedikation besprechen müssen (Tab. 2). Eine weitere Strategie zur Vermeidung von AKI in der hausärztlichen Versorgung sind die sogenannten Krankheitstag-Aktionspläne, die eine Liste von Medikamenten enthalten, die die Patienten vorübergehend absetzen sollten, wenn sie krank oder dehydriert sind. ACE-Hemmer, ARB und Diuretika können während akuter Erkrankungen vorübergehend abgesetzt werden, sollten jedoch wieder aufgenommen werden, wenn sich der Zustand stabilisiert.

Zur Verhinderung einer AKI in der Primärversorgung bei Patienten mit CKD, die krank oder dehydriert sind, sollten erkrankte Patienten, die nicht in der Lage sind, ausreichend Flüssigkeit aufzunehmen (beispielsweise aufgrund von Magen-Darm-Beschwerden), angehalten werden, Medikamente abzusetzen, die

- das Risiko eines Nierenfunktionsabfalls erhöhen (etwa ACE-Hemmer, ARB, NSAR, Diuretika, Natrium-Glukose-Kotransporter-2[SGLT-2]-Inhibitoren) und
- die Clearance reduzieren und dadurch das Risiko für UAW erhöhen (etwa Metformin und Sulfonylharnstoffe).

## Fazit für die Praxis

- Adäquate Polypharmazie kann dazu beitragen, die Lebensqualität zu verbessern, indem sie Symptome reduziert und Komplikationen verhindert.
- Inadäquate Polypharmazie führt zu Problemen wie vermehrten schweren Nebenwirkungen und Arzneimittelinteraktionen, komplexen Therapieschemata, hoher Medikamentenbelastung und einer häufigen Nichteinhaltung der Therapie.
- Polypharmazie soll durch stetige Überprüfung der Nierenfunktion, Dosisanpassung, Medikamentenauswahl und Medikamentenreduktion laufend angepasst werden.
- Eine Nutzen-Risiko-Abwägung jeder einzelnen Medikation ist regelmäßig durchzuführen.
- Eine gute Patientenaufklärung und die Sicherstellung der medizinischen und pharmazeutischen Betreuung durch Zusammenarbeit verschiedener Fachkräfte sind essenziell für eine optimale Therapie.
